# The *P. falciparum* alternative histones Pf H2A.Z and Pf H2B.Z are dynamically acetylated and antagonized by PfSir2 histone deacetylases at heterochromatin boundaries

**DOI:** 10.1128/mbio.02014-23

**Published:** 2023-10-26

**Authors:** Suffian Azizan, Shamista A. Selvarajah, Jingyi Tang, Myriam D. Jeninga, Danae Schulz, Kapil Pareek, Tamara Herr, Karen P. Day, Tania F. De Koning-Ward, Michaela Petter, Michael F. Duffy

**Affiliations:** 1 School of BioSciences, The University of Melbourne, Melbourne, Australia; 2 Bio21 Institute, Parkville, Victoria, Australia; 3 School of Medicine, Faculty of Health, Deakin University, Geelong Waurn Ponds Campus, Waurn Ponds, Australia; 4 Universitätsklinikum Erlangen, Mikrobiologisches Institut – Klinische Mikrobiologie, Immunologie und Hygiene, Friedrich-Alexander-Universität (FAU) Erlangen-Nürnberg, Erlangen, Germany; 5 Harvey Mudd College, Claremont, California, USA; 6 Department of Microbiology and Immunology, Peter Doherty Institute for Infection and Immunity, The University of Melbourne, Melbourne, Victoria, Australia; University of Geneva, Geneva, Switzerland

**Keywords:** malaria, variant histones, chromatin, epigenetics, regulation of gene expression

## Abstract

The *Plasmodium falciparum* alternative histones Pf H2A.Z and Pf H2B.Z are enriched in the same nucleosomes in intergenic euchromatin but depleted from heterochromatin. They occupy most promoters but are only dynamically associated with expression at *var* genes. In other organisms, acetylation of H2A.Z is important for its functions in gene expression and chromatin structure. Here, we show that acetylated Pf H2A.Z and Pf H2B.Z are dynamically associated with gene expression at promoters. In addition, acetylated Pf H2A.Z and Pf H2B.Z are antagonized by the sirtuin class III histone deacetylases (HDAC) PfSir2A and B at heterochromatin boundaries and encroach upon heterochromatin in parasites lacking PfSir2A or B. However, the majority of acetylated Pf H2A.Z and Pf H2B.Z are deacetylated by class I or II HDACs. Acetylated Pf H2A.Z and Pf H2B.Z are also dynamically associated with promoter activity of both canonical upstream *var* gene promoters and *var* gene introns. These findings suggest that both acetylated Pf H2A.Z and Pf H2B.Z play critical roles in gene expression and contribute to maintenance of chromatin structure at the boundaries of subtelomeric, facultative heterochromatin, critical for the variegated expression of genes that enable rapid adaptation to altered host environments.

**IMPORTANCE:**

The malaria parasite *Plasmodium falciparum* relies on variant expression of members of multi-gene families as a strategy for environmental adaptation to promote parasite survival and pathogenesis. These genes are located in transcriptionally silenced DNA regions. A limited number of these genes escape gene silencing, and switching between them confers variant fitness on parasite progeny. Here, we show that PfSir2 histone deacetylases antagonize DNA-interacting acetylated alternative histones at the boundaries between active and silent DNA. This finding implicates acetylated alternative histones in the mechanism regulating *P. falciparum* variant gene silencing and thus malaria pathogenesis. This work also revealed that acetylation of alternative histones at promoters is dynamically associated with promoter activity across the genome, implicating acetylation of alternative histones in gene regulation genome wide. Understanding mechanisms of gene regulation in *P. falciparum* may aid in the development of new therapeutic strategies for malaria, which killed 619,000 people in 2021.

## INTRODUCTION

*Plasmodium falciparum* is the pathogen responsible for the most severe forms of malaria and for the vast majority of the approximately 619,000 deaths in 2021 attributed to malaria (1). *P. falciparum* has a complex lifecycle involving two hosts and multiple morphological forms that inhabit different intra- and extra-cellular environments within these hosts. The parasite switches on transcription of suites of genes to adapt to these altered environments, and in the 48-hour asexual intra-erythrocytic cycle, the parasite expresses the majority of its genes in a transcriptional cascade that has several bursts of abundance at stage transitions (2, 3). The parasite has a relatively low number of known specific transcription factors (4, 5) and thus may rely heavily on chromatin to regulate this cascade of gene expression. The parasite maintains most of its genome in an atypically euchromatic state characterized by the enrichment of acetylated canonical histones, except in restricted heterochromatic domains at the telomeres and at several chromosome-internal sites (6).

A key feature of asexual *P. falciparum* chromatin is the intergenic enrichment in euchromatin of the alternative histones Pf H2A.Z and Pf H2B.Z and their depletion from heterochromatin (7–10). The alternative histone Pf H2A.Z is conserved throughout evolution except for its divergent N-terminal tail, while Pf H2B.Z is unique to apicomplexans. *P. falciparum* promoters are enriched with nucleosomes containing both Pf H2A.Z and Pf H2B.Z (7–10) consistent with a role for Pf H2A.Z/Pf H2B.Z in promoter structure and gene regulation. Pf H2A.Z and Pf H2B.Z levels typically do not fluctuate nor correlate with the timing of gene transcription (7–10), but promoters with the highest levels of Pf H2A.Z are the most highly transcribed at some point during the intra-erythrocytic lifecycle (7, 11). Similarly, in *Saccharomyces cerevisiae,* high levels of H2A.Z at promoters are required for expression of many genes despite an anti-correlation with temporal transcription (12). These findings suggest an evolutionarily conserved role for H2A.Z in defining the potential of regulatory regions to drive gene expression.

H2A.Z plays diverse roles in chromatin structure, heterochromatin maintenance, mitosis, DNA repair, and gene expression. Contradictory effects on gene expression have been reported for H2A.Z, consistent with differing reports on its effects on nucleosome stability (13–15), but in the most recent cryo-EM study, H2A.Z incorporation decreased nucleosome stability (15). Inactive human promoters are enriched in H2A.Z, which is evicted upon gene induction (14). The position of H2A.Z nucleosomes within promoters seems to be critical for their effect upon gene expression. Poorly positioned H2A.Z nucleosomes are associated with gene repression, whereas a strongly positioned +1 H2A.Z-containing nucleosome is associated with high levels of gene expression (16). Acetylation of metazoan H2A.Z reduces nucleosome stability (17), and destabilization is markedly increased when other histones in the nucleosome are also acetylated (18). As chicken, yeast, and human promoters as well as human enhancers are activated, their acetylated H2A.Z levels increase, and their levels of unmodified H2A.Z decrease (19–24). These observations are consistent with a model where unmodified H2A.Z-containing nucleosomes mark promoters that are then activated when the nucleosomes are destabilized by H2A.Z acetylation. How the destabilized H2A.Z is evicted from active promoters remains unclear, but the pre-initiation complex (PIC) itself is required to remove H2A.Z from nucleosomes at promoters (25), and the ATP-dependent chromatin remodeler INO80 can distort DNA to preferentially replace H2A.Z with H2A in yeast (26). The N-terminal tails of Pf H2B.Z and Pf H2A.Z are heavily acetylated (27–31), which suggests that these acetylated *P. falciparum* alternative histones may play a similar, important, and functional role in *P. falciparum* gene regulation.

Recently, a number of novel regulatory complexes were identified through affinity of their bromodomain protein (BDP) components for different *P. falciparum* histone acetylations (32). The combinatorial interactions between these complexes and the specific transcription factors would go some way toward explaining the tightly regulated transcriptional cascade of otherwise euchromatic genes. For example, the specific transcription factor AP2-I and the bromodomain protein PfBDP1 between them coordinate expression of a subset of erythrocyte invasion genes, but both factors also function independently to regulate other genes (33, 34). PfBDP1 interacts with two other bromodomain proteins, PfBDP2 and PfBDP7 (32, 33, 35), and the complex binds strongly to multi-acetylated peptides derived from the alternative histones Pf H2A.Z and Pf H2B.Z (32).

The restricted islands of *P. falciparum* facultative heterochromatin that are depleted of Pf H2A.Z/Pf H2B.Z nucleosomes in asexual parasites are enriched in multi-gene families including the *var* genes that encode the immunodominant, variant antigen, and cytoadhesin PfEMP1 (8). A single *var* gene is expressed at one time in a process dependent upon silencing of the rest of the *var* repertoire by the PfSir2 sirtuin and PfHda2 histone deacetylases (36–39). Uniquely among *P. falciparum* genes, the *var* gene promoters are dynamically enriched in Pf H2A.Z/Pf H2B.Z nucleosomes in association with *var* gene expression (8). *Var* gene introns also have a regulatory function driving the expression of regulatory ncRNAs (40, 41) and are also enriched in Pf H2A.Z/Pf H2B.Z nucleosomes (8, 10); however, this is unlinked to the activity of the upstream promoter. The removal of Pf H2A.Z/Pf H2B.Z nucleosomes from transiently repressed *var* gene promoters is dependent on the histone deacetylase PfSir2A (8). In *S. cerevisiae,* H2A.Z plays a crucial role for genomic chromatin structure by antagonizing the spread of Sir2-dependent telomeric heterochromatin (42, 43), and it also antagonizes sirtuin-dependent gene silencing (44). It is plausible that Pf H2A.Z/Pf H2B.Z nucleosomes play a similar important role in confining *P. falciparum* facultative heterochromatin.

Here, we show that acetylated Pf H2A.Z (Pf H2A.Zac) and acetylated Pf H2B.Z (Pf H2B.Zac) are dynamically associated with gene expression across the *P. falciparum* genome. This suggests that Pf H2A.Z/Pf H2B.Z nucleosomes help define promoters and that their acetylation could recruit transcription regulating complexes or destabilize the nucleosomes leading to activation of the underlying promoter. We also show that the ablation of PfSir2A results in nucleosomes containing Pf H2A.Zac/Pf H2B.Zac encroaching on heterochromatin boundaries, suggesting that at sites of facultative heterochromatin, sirtuins antagonize the alternative histones and their acetylation. However, ablation of PfSir2A or PfSir2B has no significant effect on the alternative histones beyond the heterochromatin boundaries with the distribution of both acetylated and total Pf H2A.Z and Pf H2B.Z unaffected in euchromatin and still largely excluded from within the facultative heterochromatin domains. Pf H2A.Zac and Pf H2B.Zac are also enriched in introns of *var* genes in association with sterile second exon transcripts. Overall, these patterns suggest that Pf H2A.Zac and Pf H2B.Zac play a critical role in establishing functional chromatin structure in these subtelomeric regions.

## RESULTS

### Generation and characterization of Pf H2A.ZK11K15ac- and Pf H2B.ZK3K8ac-specific antibodies

**Fig 1:**
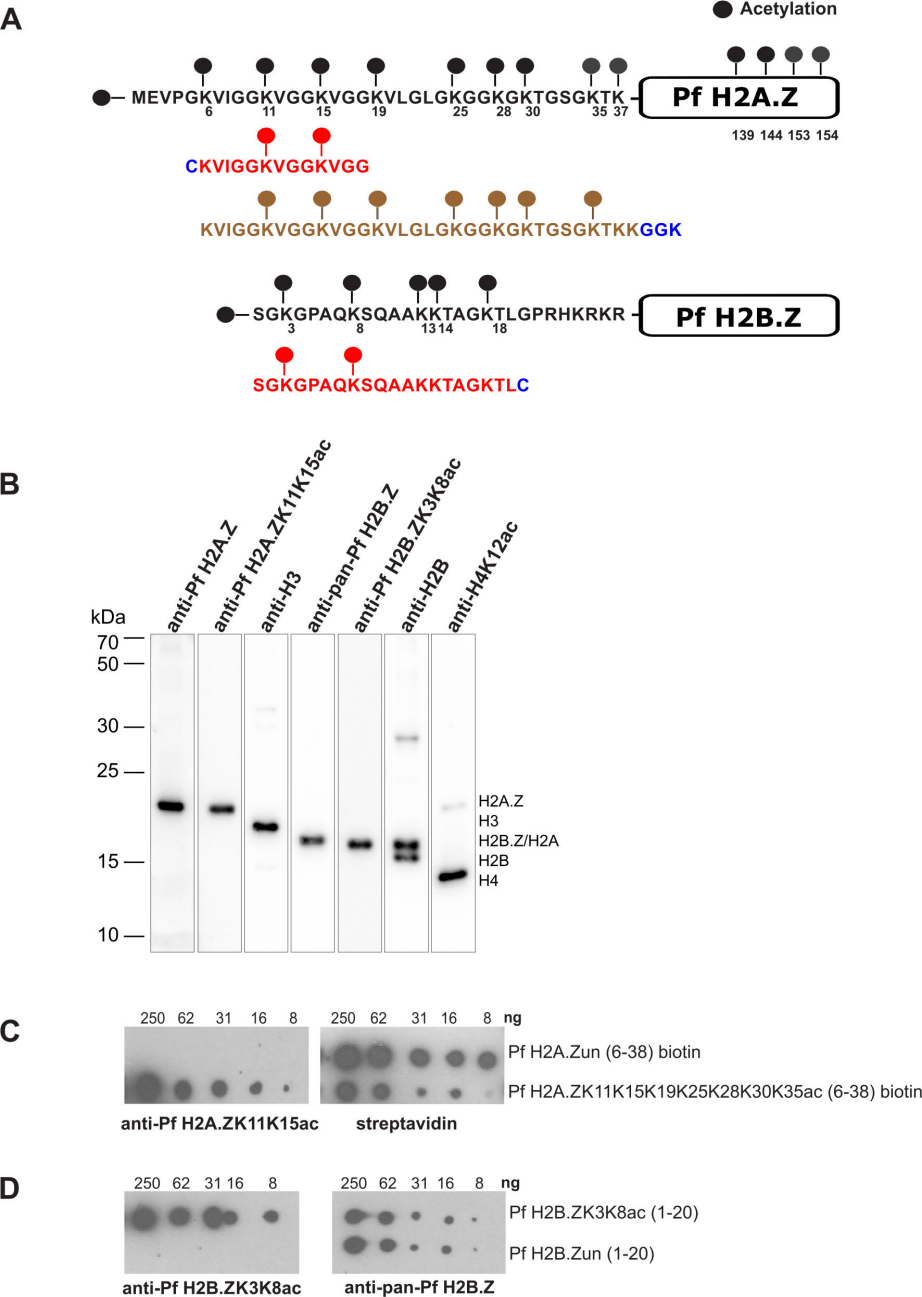
Validation of antibodies specifically targeting acetylated Pf H2A.Z and Pf H2B.Z. (**A**) Diagram showing the known acetylations of the N terminal tails of Pf H2A.Z and Pf H2B.Z with the acetylated peptides used to immunize rabbits in red below and the multi-acetylated biotinylated Pf H2A.Z peptide used for a dot blot in brown. The peptides included terminal cysteine or GGK residues (in blue) for conjugation to keyhole limpet cyanin or biotin, respectively. (**B**) Western blot of parasite extract probed with our previously validated rabbit α-Pf H2A.Z antiserum (8), affinity-purified α-H2A.ZK11K15ac antibody, α-H3 (Abcam, Ab1791), α-Pf H2B.Z antibody, α-Pf H2B.ZK3K8ac antibody, α-H2B antibody (Abcam, Ab1790), and α-H4K12ac antibody, which cross-reacts with Pf H2A.Z (Millipore, 07–595). (**C–D**) The unconjugated acetylated and unacetylated peptides were titrated on dot blots and probed with the affinity-purified α-Pf H2A.Zac or α-Pf H2B.Zac antibodies and the α-Pf H2B.Zac pan reactive antibody. (**C**) Dotblot of titrated biotinylated Pf H2A.Z multi-acetylated peptide and matching unacetylated peptide probed with α-Pf H2A.ZK11K15ac and streptavidin. (**D**) Dotblot of titrated unconjugated Pf H2B.ZK3K8ac and matching unacetylated peptide probed with purified Pf H2B.ZK3K8ac antibody and the anti-pan-Pf H2B.Z antibody.

Pf H2A.Z and Pf H2B.Z are acetylated at multiple sites in their N-terminal tails (27–31) . To study the function of variant histone acetylation in *P. falciparum*, we designed peptides that cover non-redundant acetylated epitopes in the N-terminal tails of the two variant histones (Fig. 1A). Some acetylated epitopes occurring in Pf H2A.Z are also present in H4 (LGKacGG and GKacGGKacG motifs), as evident from the cross-reactivity of H4K12ac antibodies with Pf H2A.Z (8) (Fig. 1B). Therefore, the region L21 to G29 was avoided, and a peptide covering Pf H2A.Z amino acids 6–18 and containing two repeats of the Pf H2A.Z-specific GGKacVGG epitope (Pf H2A.ZK11K15ac) was synthesized. Pf H2B.Z shares an AKKT motif with H2B covering K13 and K14, so a peptide covering the Pf H2B.Z-specific N-terminal acetylation sites at K3 and K8 was synthesized (Pf H2B.ZK3K8ac). Both peptides were conjugated to keyhole limpet hemocyanin and used to immunize rabbits (Anaspec) (Fig. 1A). To isolate acetyl-specific anti-Pf H2A.ZK11K15ac and anti-Pf H2B.ZK3K8ac antibodies, the rabbit antisera were incubated with immobilized acetylated peptides, and the bound antibodies were eluted and cross-adsorbed against immobilized, non-acetylated peptides. Pan-reactive antibodies able to recognize both acetylated and unacetylated forms of Pf H2B.Z variants were then obtained from the fraction bound to the non-acetylated peptides (anti-pan-Pf H2B.Z). As the anti-pan-Pf H2A.Z antibody did not detect the unacetylated Pf H2A.Z peptides (not shown), we instead used an anti-Pf H2A.Z antiserum in this study, which we had previously shown bound both unacetylated and acetylated forms of Pf H2A.Z (8).

The specificity for alternative histones of antibodies to acetylated Pf H2A.Z or Pf H2B.Z or to anti-Pf H2B.Z was confirmed using Western blots of *P. falciparum* histone extract (Fig. 1B). Moreover, the specificities of the purified anti-Pf H2A.Zac and anti-Pf H2B.Zac antibodies for the acetylated forms of Pf H2A.Z and Pf H2B.Z, and of the purified anti-pan-Pf H2B.Z antibodies for both acetylated and unacetylated Pf H2B.Z were confirmed by probing dot blots of unconjugated acetylated and unacetylated peptides of the same sequence as used for immunization (Fig. 1C and D). The anti-Pf H2A.Zac and anti-Pf H2B.Zac antibodies also did not react with a range of singe- and multiple-acetylated H3 and H4 peptides by dot blot (Fig. S1).

### The location of acetylated Pf H2A.Z and Pf H2B.Z correlates with the location of total Pf H2A.Z and Pf H2B.Z

*P. falciparum* wild-type 3C parasites that were selected from 3D7 to predominantly express the *var2csa* gene (45) were analyzed by cross-linked chromatin immunoprecipitation followed by DNA sequencing (ChIPseq) to identify genomic sites where Pf H2A.Z and Pf H2B.Z were acetylated. In both ring- and schizont-stage parasites, Pf H2A.Z and Pf H2B.Z were highly correlated. Pf H2A.Zac and Pf H2B.Zac were highly correlated in schizonts and moderately correlated in ring stages due to variation between the Pf H2B.Zac ring-stage replicates (Fig. S2A). These observations were consistent with the published exclusive co-occupancy of nucleosomes by Pf H2A.Z and Pf H2B.Z and further suggested that if one variant was acetylated, the other likely was as well. In both ring and schizont parasites, the total levels of each alternative histone also correlated well with the levels of its acetylated forms (all spearman *r* >0.62) (Fig. S2A and B), suggesting that at most sites where Pf H2A.Z and Pf H2B.Z were present, some of the Pf H2A.Z and Pf H2B.Z were acetylated.

### Acetylated Pf H2A.Z and Pf H2B.Z are enriched in intergenic euchromatin

**Fig 2:**
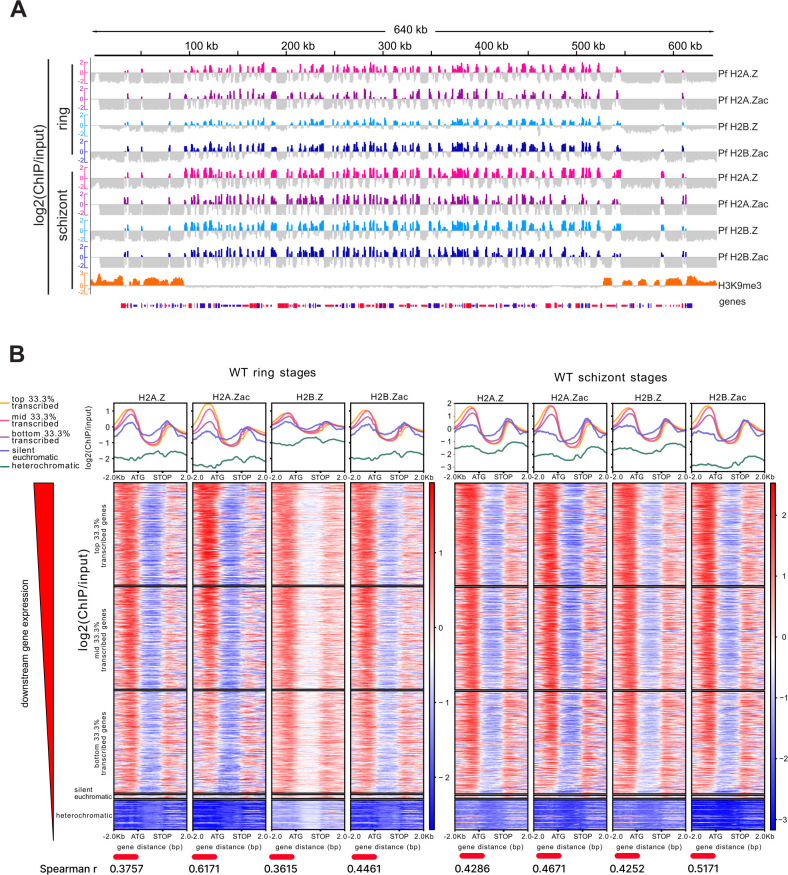
Alternative histones and their acetylated forms are enriched in upstream, intergenic sequence. (**A**) Log_2_(ChIP/input) of Pf H2A.Z, Pf H2B.Z, and their acetylated forms plotted across chromosome 1 for chondroitin sulfate A selected 3C ring- and schizont-stage parasites. H3K9me3 in parental 3D7 schizont stages is included as a marker of heterochromatin. (**B**) Average profile plots and corresponding heatmaps of log_2_(ChIP/input) for the alternative histones and their acetylated forms in 3C ring- and schizont-stage parasites plotted from 2 kb upstream to 2 kb downstream of all *P. falciparum* genes in descending order of expression level [fragments per kilobase of exon per million reads (FPKM)]. Genes were grouped into the five groups of heterochromatic genes; silent euchromatic genes (0 FPKM); and top, mid, and bottom terciles of expressed euchromatic genes. The Spearman *r* correlation between the log_2_(Chip/input) within the 2-kb region upstream of a gene and the gene’s expression (FPKM) is shown under each heatmap (all *P* < 2 × 10^−152^).

We used H3K9me3 broad peaks called from 3D7 ChIPseq (46) to define the heterochromatic regions. In wild-type ring- and schizont-stage parasites, Pf H2A.Zac and Pf H2B.Zac were enriched in euchromatin and largely excluded from the H3K9me3-enriched heterochromatin (Fig. 2A). Pf H2A.Zac and Pf H2B.Zac were primarily enriched in intergenic regions (Fig. 2B), consistent with the known occupancy pattern of total Pf H2A.Z and Pf H2B.Z (7–10). The intergenic enrichment was predominantly upstream of genes and was positively correlated with the level of gene expression (Fig. 2B), as previously observed for Pf H2A.Z (47). Interestingly, total and acetylated Pf H2A.Z and Pf H2B.Z were also more enriched in the coding sequence of silent euchromatic genes relative to the coding sequence of expressed euchromatic genes (Fig. 2B), similar to *Toxoplasma gondii* (48).

### Acetylated Pf H2A.Z and Pf H2B.Z are dynamically enriched at active gene promoters

**Fig 3:**
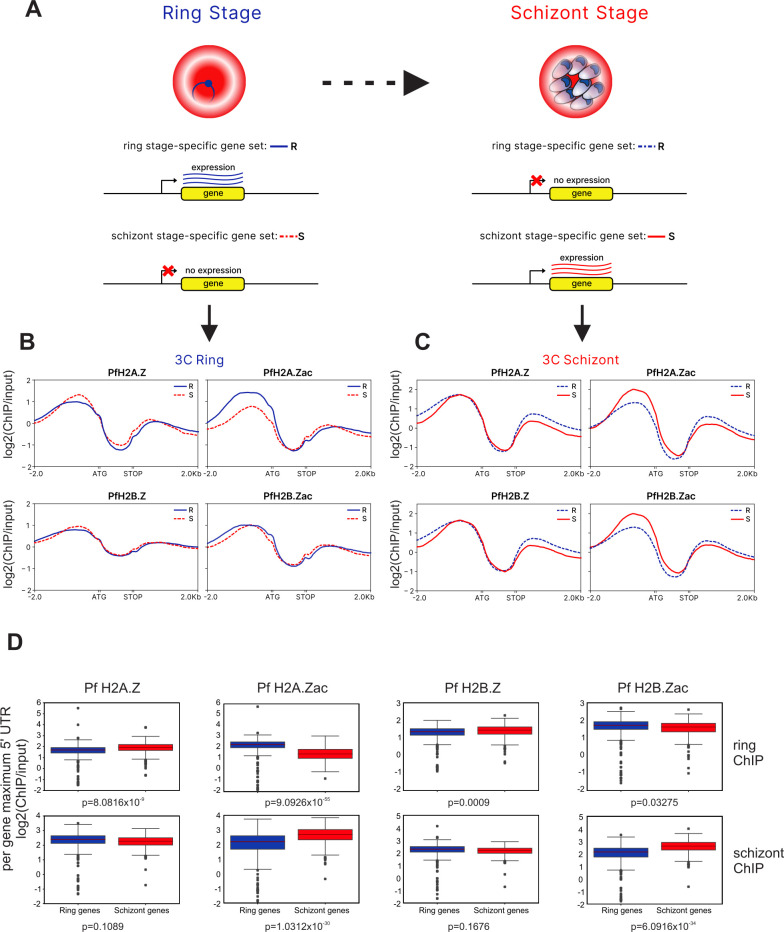
Pf H2A.Z and Pf H2B.Z are dynamically enriched at promoters of expressed genes. (**A**) Strategy for selecting stage-specific expressed gene sets that were in the top quartile of expression in a stage and were expressed at least 1.5-fold higher in that stage than the other stage. (**B, C**) Log_2_(ChIP/input) average profiles of ring (blue) or schizont (red) stage-expressed gene sets from (**B**) ring- and (**C**) schizont-stage ChIPs of wild-type parasites [ring-stage-expressed gene set (*n* = 675) and schizont-stage-expressed gene set (*n* = 392)]. Within a stage, the upregulated gene sets are solid lines, and the downregulated gene sets are dashed lines. (**D**) Log_2_(ChIP/input) maximum values for 50 bp windows across 2 kb upstream of genes compared between the same ring and schizont dynamically expressed genes sets from (B and C) for each total and acetylated alternative histone ChIP, *P* values Wilcoxon signed-rank test.

Upstream enrichment patterns of Pf H2A.Z and Pf H2B.Z were positively correlated with transcript levels (Fig. 2), but they were not dynamically associated with gene expression throughout the asexual intra-erythrocytic lifecycle in previous studies (7–10). This indicated that the alternative histones help define the strength of promoters rather than their temporal regulation (9). However, the upstream levels of acetylated Pf H2A.Z and Pf H2B.Z had a stronger association with gene expression than did total levels of Pf H2A.Z and Pf H2B.Z (Fig. 2B). To investigate whether acetylated alternative histones were dynamically enriched at stage-specific expressed genes, we compared enrichment patterns of total alternative histones and their acetylated forms across the promoters of dynamically regulated genes in ring- and schizont-stage parasites. Matched RNAseq data from the same cultures used for ChIPseq (Fig. S2E) were analyzed to select sets of genes that were in the top quartile by expression in either rings or schizonts and were expressed at least 1.5-fold more in one lifecycle stage than in the other stages (Fig. 3A). The resulting stage-specific gene sets contained 675 genes for ring stage and 392 genes for schizont-stage wild-type parasites (Fig. 3A). Average log_2_(ChIP/input) from the concatenated ChIPseq replicates was plotted for the gene sets. There were subtle depletions in 5′ intergenic enrichment of total Pf H2A.Z or total Pf H2B.Z in the dynamically expressed gene sets compared to the dynamically repressed gene sets in rings and schizonts (Fig. 3B and C). However, both Pf H2A.Zac and Pf H2B.Zac were clearly dynamically enriched upstream of genes expressed in schizonts (Fig. 3C). In ring stages, Pf H2A.Zac was also clearly dynamically enriched upstream of expressed genes but Pf H2B.Zac was not (Fig. 3B).

To confirm the significance of the dynamic association between acetylated Pf H2A.Z and Pf H2B.Z and gene expression, the 2-kb sequence upstream of the stage-specific gene sets was examined in each ChIP data set to identify the 50-bp window with maximum ChIP/input value, and those maximum values were compared (Fig. 3D). This approach should center on +1 nucleosomes at the transcriptional start site (TSS) rather than diluting the signal from the TSS by averaging enrichment across the 2-kb 5′ untranslated region (UTR). For ChIPs of both rings and schizonts, acetylated Pf H2A.Z and acetylated Pf H2B.Z levels were significantly higher upstream of dynamically expressed than dynamically repressed genes (Wilcoxon signed-rank test) (Fig. 3D). Interestingly, in ring stages, the maximum levels of total Pf H2A.Z and total Pf H2B.Z were slightly but significantly lower in dynamically expressed genes than in dynamically repressed genes, and the same trend was evident, although not significant, in schizonts (Fig. 3D). This is consistent with reports from some organisms that H2A.Z was evicted from active gene promoters.

### PfSir2A and PfSir2B antagonize Pf H2A.Z and Pf H2B.Z at chromatin boundaries

**Fig 4:**
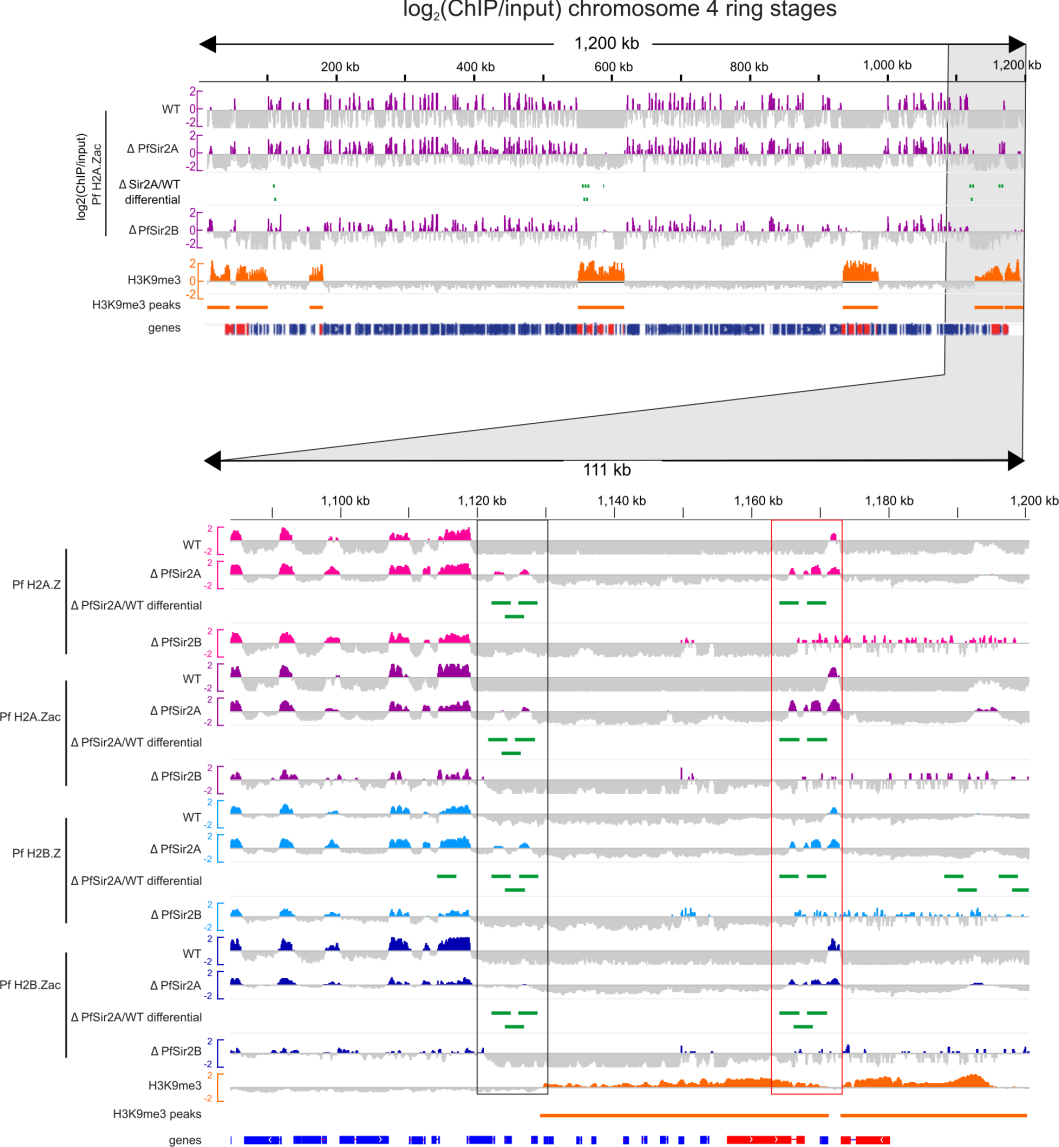
Total and acetylated Pf H2A.Z and Pf H2B.Z are differentially enriched in heterochromatin in *∆PfSir2A* and *∆PfSir2B* parasites compared to wild-type (WT) ring stages. A projection of chromosome 4 in ring-stage wild-type 3C, *∆PfSir2A,* and *∆PfSir2B* parasites showing Pf H2A.Zac log_2_(ChIP/input) levels (purple); peaks of Pf H2A.Zac identified as differentially enriched in *∆PfSir2A* compared to wild type (*∆PfSir2A*/WT differential, green); ring-stage log_2_(ChIP/input) H3K9me3 (orange); and peaks of H3K9me3 in wild-type 3D7 (orange lines). Genes are indicated in blue, with *var* genes superimposed in red. The lower panel zooms into 111 kb from the right-hand end of chromosome 4 and shows the data as above but also for total and acetylated Pf H2A.Z and Pf H2B.Z with boxed regions indicating regions of differential enrichment of total and acetylated histones between wild-type and *∆PfSir2A* parasites at the euchromatin/heterochromatin boundary (black box) and downstream of upsB *var* genes within subtelomeric heterochromatin domains (red box).

Because PfSir2A dynamically antagonizes Pf H2A.Z at active *var* gene promoters (8) and H2A.Z antagonizes telomeric Sir2 in *S. cerevisiae* (42), we next investigated Pf H2A.Z, Pf H2B.Z, and their acetylations in *∆PfSir2A* and *∆PfSir2B* parasites (36, 38). Overall, the patterns of enrichment of Pf H2A.Zac and Pf H2B.Zac were similar in the wild-type parasites and the *∆PfSir2A* or *∆PfSir2B* parasites (Fig. 4). This is consistent with the predicted function of PfSir2A and B in specifically maintaining heterochromatin (36–38), a region from which Pf H2A.Z and Pf H2B.Z are depleted. To investigate whether more subtle differences in alternative histone enrichment occurred at the euchromatin/heterochromatin boundaries, total or acetylated Pf H2A.Z and Pf H2B.Z ChIPseq peaks were identified by MACS2 (49), and peaks that differed significantly between the wild-type and *∆PfSir2A* parasites were identified by CSAW (50).

In the *∆PfSir2A* ring- and schizont-stage parasites, Pf H2A.Z and Pf H2B.Z and their acetylated forms were differentially enriched within heterochromatin as defined by MACS2 called peaks of H3K9me3 in 3D7 ChIPseq (46). They were also enriched in euchromatin adjacent to the boundaries with heterochromatin (Fig. 4; Fig. S3). In the *∆PfSir2B* parasites, total and acetylated Pf H2A.Z and Pf H2B.Z appeared more enriched in telomeric and subtelomeric heterochromatin regions (Fig. 4; Fig. S3).

**Fig 5:**
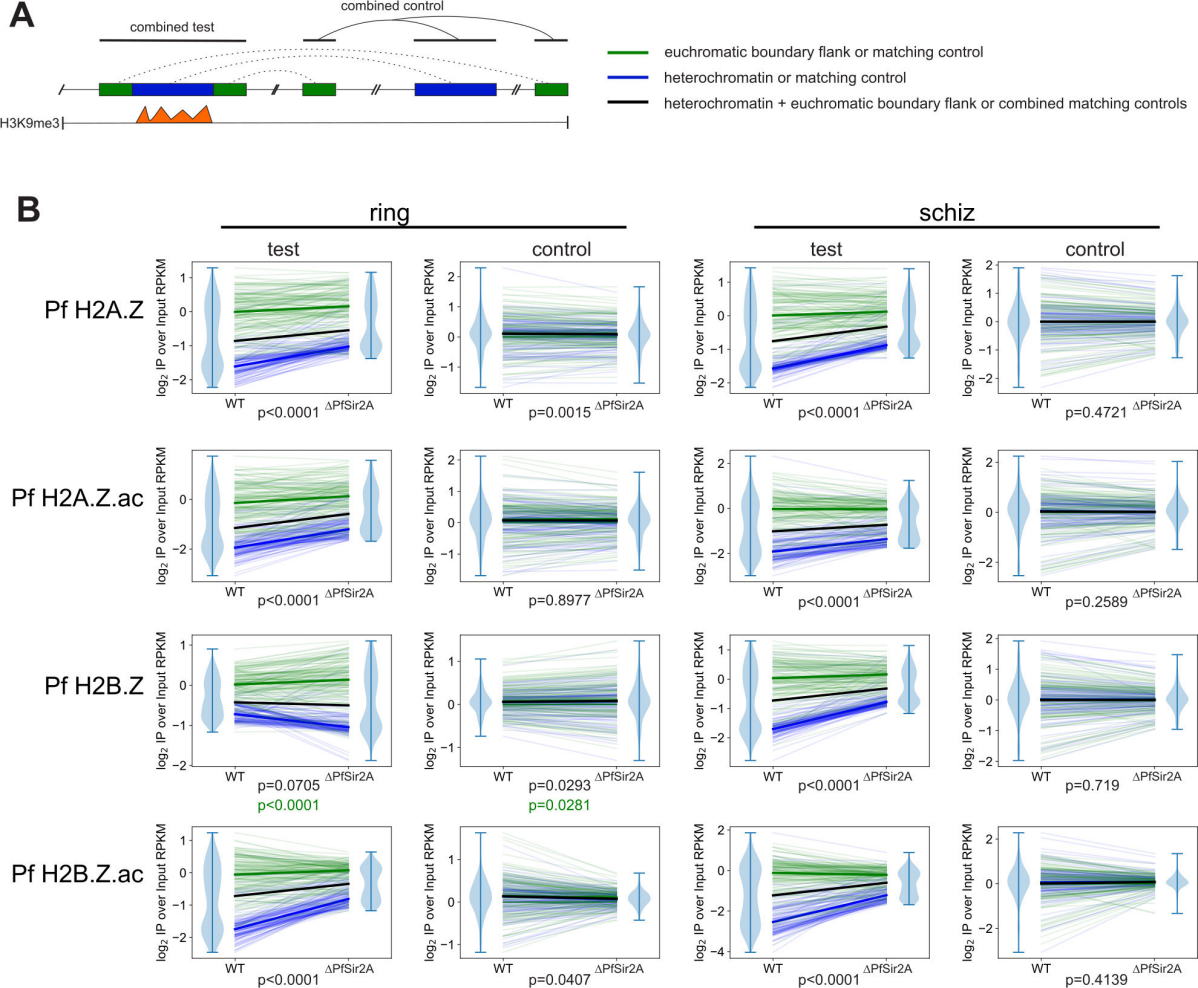
Total and acetylated Pf H2A.Z and Pf H2B.Z are antagonized by PfSir2A at heterochromatin boundaries. (**A**) Model of scheme for selecting test and control regions for comparison between wild-type (WT) and *∆PfSir2A* parasites. The compared regions were 109 ring-stage and 100 schizont-stage heterochromatic test regions defined by H3K9me3 broad peaks called from 3D7 ChIPseq (blue) (46) and 138 ring-stage and 131 schizont-stage non-overlapping, flank test regions, each of up to 10 kb of sequence (green). Size- and chromosome-matched control sequences were randomly selected (dotted lines) for each test sequence and are color coded the same. (**B**) Average ratios of log_2_(ChIP/input) for two replicates of total and acetylated Pf H2A.Z and Pf H2B.Z ChIP normalized as RPKM compared between wild-type and *∆PfSir2A* parasites. Medians for heterochromatin and matched controls (blue) or flanks and matched controls (green) are shown in thick, bold lines, and medians for the combined heterochromatin and flank test regions or their matching controls are shown in thick black bold. *P* values are for Wilcoxon matched-pairs signed-rank test comparisons between wild-type and *∆PfSir2A* parasites of combined heterochromatin and flank test regions or their matching controls (in black) or flanking regions only (in green for Pf H2B.Z). Violin plots at the edges of line plots indicate the frequency distribution of the combined heterochromatin and flank regions or matching control regions, and bars indicate the range.

To test whether Pf H2A.Z and Pf H2B.Z and their acetylated forms were indeed selectively enriched at the heterochromatin-euchromatin boundaries in *∆PfSir2A* and *∆PfSir2B* compared to wild-type parasites, we defined the entire H3K9me3 broad peaks called from 3D7 ChIPseq (46) as 109 ring-stage and 100 schizont-stage heterochromatic test regions. In addition, 138 ring-stage and 131 schizont-stage non-overlapping, flank test regions, each of up to 10 kb of sequence that flanked the H3K9me3 peaks were also identified. Randomly selected size- and chromosome-matched control sequences were selected for each test sequence from the genome masked for the test sequences. Flank and peak read counts were normalized as reads per kilobase per million reads (RPKM), and the average ratio of ChIP/input for two replicates was compared between wild-type and *∆PfSir2A* (Fig. 5) or ∆*PfSir2B* parasites (Fig. S4). In both, rings and schizonts, there were significantly higher levels of Pf H2A.Z, Pf H2A.Zac, and Pf H2B.Zac in the heterochromatin and flanking sequences in the *∆PfSir2A* and ∆*PfSir2B* parasites compared to wild-type parasites (Wilcoxon matched-pairs signed-rank test all *P* < 0.0001) (Fig. 5; Fig. S5). The median values for control sequences were visually indistinguishable between wild-type and *∆PfSir2A* or ∆*PfSir2B* parasites, but in ring stages, the levels of Pf H2B.Zac and Pf H2A.Z in controls trended in the opposite direction to the tests and were actually slightly less in the *∆PfSir2A* or ∆*PfSir2B* parasites compared to wild-type parasites (*P* ≤ 0.0407) (Fig. 5; Fig. S4).

In schizonts, Pf H2B.Z also increased in heterochromatin and flanking regions in *∆PfSir2A* and ∆*PfSir2B* parasites compared to wild-type parasites (Fig. 5; Fig. S4). In ring stages, the levels of Pf H2B.Z decreased in heterochromatin in both *∆PfSir2A* and ∆*PfSir2B* parasites compared to wild-type parasites but increased in euchromatic flanking regions in *∆PfSir2A* (*P* < 0.0001) but not ∆*PfSir2B* parasites compared to wild-type parasites. The control flanking regions in rings also had a modest increase in Pf H2B.Z levels in *∆PfSir2A* compared to wild type (*P* = 0.0281), preventing any clear inference of an association between PfSir2A and the deposition of Pf H2B.Z in and around heterochromatin in ring-stage parasites. Overall, however, we concluded that Pf H2A.Z, Pf H2A.Zac, Pf H2B.Z, and Pf H2B.Zac were antagonized in heterochromatin and at heterochromatin boundaries by the histone deacetylases PfSir2A and PfSir2B.

### Acetylated Pf H2A.Z and Pf H2B.Z are enriched downstream of ups B *var* genes

**Fig 6:**
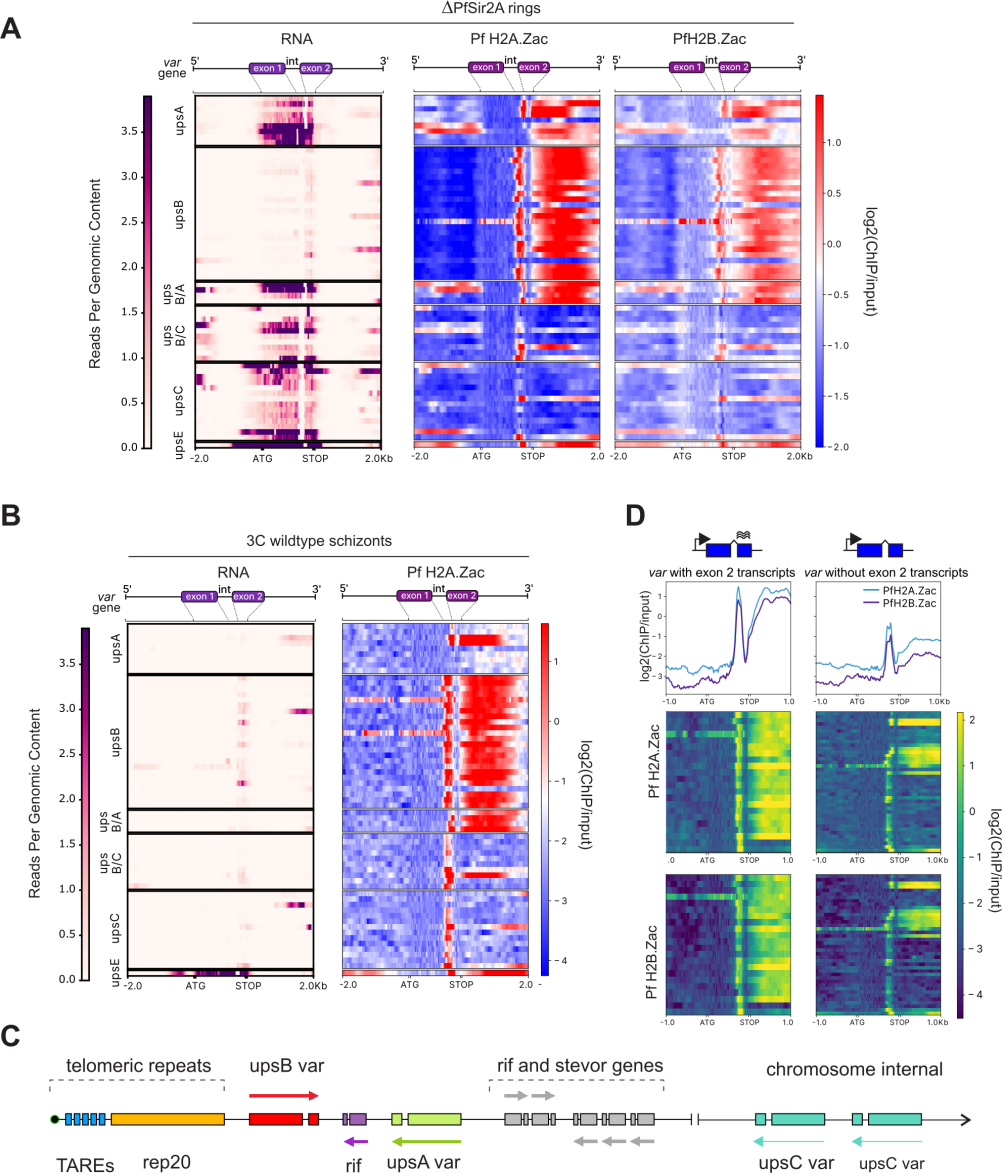
Pf H2A.Zac and Pf H2B.Zac are enriched at transcribed *var* gene promoters and *var* introns. RNAseq reads per genomic content and log_2_(ChIP/input) for Pf H2A.Zac and Pf H2B.Zac plotted from 2 kb upstream to 2 kb downstream of the genomic repertoire of 65 *var* genes in (**A**) ring-stage *∆PfSir2A* parasites and (**B**) schizont-stage wild-type parasites. The heatmaps are scaled to render all *var* genes the same length; therefore, the gene diagrams at the top of the heatmaps only approximate the intron-exon boundaries (int, intron). (**C**) Diagram illustrating the genomic arrangement of *var* genes in subtelomeric and chromosome internal heterochromatin. (**D**) Log_2_(ChIP/input) of Pf H2A.Zac and Pf H2B.Zac across *var* genes that did or did not transcribe the exon 2 in schizont-stage wild-type parasites. TAREs = telomere associated repeat elements.

Pf H2A.Z and Pf H2B.Z are typically depleted at silent members of the heterochromatic *var* multigene family (8, 10), but there was a striking enrichment of total and acetylated alternative histones in the intergenic regions downstream of upsB *var* genes that was not associated with their expression (Fig. 6A and B; Fig. S5). UpsB *var* genes are the most telomeric *var* genes and are transcribed toward the centromere (Fig. 6C); thus the enrichment of acetylated canonical and alternative histone lies between the central chromosome body and the upsB *var* gene/telomere (Fig. 6C). This suggests the acetylated histones could play a structural role at this location, possibly contributing to the barrier against the spread of telomeric heterochromatin into the chromosome bodies.

### Pf H2A.Z and Pf H2B.Z are acetylated when present at *var* promoters in rings

Unlike most other genes, the levels of total Pf H2A.Z and Pf H2B.Z at *var* gene promoters are dynamically associated with *var* gene expression (8, 10). The histone deacetylase PfSir2A represses *var* genes and antagonizes Pf H2A.Z at *var* gene promoters (8, 36). To investigate the role of PfSir2A in the unique dynamics of Pf H2A.Z and Pf H2B.Z at *var* gene promoters, we assessed the histones’ acetylation at the promoters of *var* genes that were derepressed in ring-stage *∆PfSir2A* parasites (Fig. 6A). The levels of total and acetylated Pf H2A.Z and Pf H2B.Z were more highly correlated at *var* gene promoters (all *var* 2-kb 5′ untranslated regions) (Spearman *r* = 0.97) than throughout the genome ( Fig. S2) (Spearman *r* ≤0.79), suggesting that most of the Pf H2A.Z and Pf H2B.Z present at the derepressed *var* promoters were acetylated. This implicates PfSir2A deacetylase activity in Pf H2A.Z and Pf H2B.Z dynamics at *var* gene promoters.

### *Var* gene intron promoter activity is associated with enrichment of acetylated Pf H2A.Z and Pf H2B.Z

*Var* gene introns have promoter activity that has been previously associated with *var* gene silencing (40, 51). Schizont stage parasites express sterile *var* exon 2 transcripts driven by the intron promoters, primarily from upsB *var* genes (Fig. 6B and C). The presence of acetylated Pf H2A.Z and Pf H2B.Z was associated with the level of expression of sterile *var* exon 2 transcripts in schizonts (Fig. 6D; Fig. S5). This suggested that acetylated Pf H2A.Z and Pf H2B.Z also contributed to regulating *var* intron promoter activity.

### Pf H2A.Zac and Pf H2B.Zac are deacetylated by class I and II histone deacetylases

PfSir2A could potentially deacetylate Pf H2A.Z and Pf H2B.Z but only within heterochromatin and at heterochromatin boundaries (Fig. 4 and 5). Some other enzymes must therefore be responsible for the dynamic deacetylation of the bulk of Pf H2A.Zac and Pf H2B.Zac at euchromatic genes when their expression decreases. *P. falciparum* has five HDACs, the class I PfHDAC1, the class II PfHDAC2 and PfHDAC3, and the class III sirtuins, PfSir2A and PfSir2B. To investigate whether class I or II HDACs can also deacetylate Pf H2A.Zac and Pf H2B.Zac, we tested the pan-class I and II HDAC inhibitors apicidin and trichostatin A (52, 53). Treatment of parasites with both inhibitors increased the amount of acetylated Pf H2A.Z and Pf H2B.Z (Fig. S6). This suggests that the bulk of Pf H2A.Zac and Pf H2B.Zac within euchromatin is deacetylated by class I or II HDACs.

## DISCUSSION

Total and acetylated Pf H2A.Z and Pf H2B.Z have highly correlated distributions in both ring- and schizont-stage parasites (Fig. S2), suggesting that the majority of Pf H2A.Z and Pf H2B.Z are acetylated to some extent in asexual blood stage *P. falciparum*. However, upstream of genes, the levels of acetylated Pf H2A.Z and Pf H2B.Z are dynamically and positively associated with the genes’ expression. The role of H2A.Z at promoters is enigmatic, it functions differently depending on species, chromatin context, and genes studied (54). In human cells, different patterns of H2A.Z enrichment in promoters had different effects on gene expression (16). A well-positioned, micrococcal nuclease-sensitive, i.e., relatively unstable, H2A.Z-containing nucleosome at the TSS was associated with highly expressed genes, and poorly positioned, dispersed H2A.Z-containing nucleosomes were associated with gene repression. *P. falciparum* has poorly positioned nucleosomes across its promoters consistent with the slight, negative association between dynamic gene expression and total Pf H2A.Z and Pf H2B.Z enrichment in ring stages. However, there is a well-positioned nucleosome at the TSS which is frequently evicted when *P. falciparum* genes are expressed (55), consistent with the presence of an unstable nucleosome containing acetylated Pf H2A.Z.

The dynamic enrichment of acetylated Pf H2A.Z and Pf H2B.Z at active promoters could function to drive transcription through biophysical alteration of chromatin. Nucleosomes at the +1 site can block RNA PolII progression, but H2A.Z at the +1 nucleosome is important in preventing stalling of RNA PolII, suggesting that H2A.Z plays an important role in elongation, possibly through destabilizing the nucleosomes at the +1 position (56). Homotypic H2A.Z-H2A.Z nucleosomes are less stable than H2A-containing nucleosomes (57) and are enriched immediately downstream of the TSS of active genes at the +1 position (58). The only detectable Pf H2A.Z nucleosomes are homotypic (10) so are presumably less stable than H2A-containing nucleosomes. Mechanistically, the flexible DNA ends of nucleosomes containing H2A.Z reduce their stability compared to H2A-containing nucleosomes (15). Acetylation of H2A.Z reduces nucleosome stability further (17) and has been associated with promoter activity in other species (19, 23). Thus, the enrichment of acetylated Pf H2A.Z and Pf H2B.Z at promoters could destabilize nucleosomes to create chromatin that is permissive for transcription. The slightly decreased levels of total Pf H2A.Z and Pf H2B.Z upstream of dynamically expressed genes suggest that the increased levels of Pf H2A.Z and Pf H2B.Z acetylation destabilize the nucleosomes leading to their increased eviction at active gene promoters, similar to yeast and metazoans (19–24). The mechanism of H2A.Z eviction remains unclear. Both the PIC (25) and the ATP-dependent chromatin remodeler INO80 have been implicated (26), although *P. falciparum* possesses no annotated orthologs of INO80 (PlasmoDB).

Acetylated H2A.Z may recruit other factors which affect chromatin function. Pf H2A.Z co-localizes with the bromodomain protein PfBDP1 at nucleosome-depleted regions that bind the transcription factor AP2-I in expressed invasion gene promoters (11), and PfBDP1 binds acetylated Pf H2A.Z and Pf H2B.Z peptides (32), suggesting that Pf H2A.Zac may directly recruit bromodomain protein containing transcriptional complexes. Acetylated Pf H2A.Z/ Pf H2B.Z might also interact with other histone modifications and histone modifiers to affect gene expression. Incorporation of H3.3 destabilizes H2A.Z-containing nucleosomes at promoters and enhancers of active genes in vertebrates (59, 60), and Pf H3.3 is also enriched at active *var* gene promoters along with Pf H2B.Z and Pf H2A.Z (8, 10, 61). However, it does not appear to be a genome-wide mechanism for coordinating gene expression because Pf H3.3 enrichment at promoters genome wide does not correlate with *P. falciparum* gene expression (61).

*P. falciparum* relies on variegated expression of contingency genes as a bet-hedging strategy for environmental adaptation (62). These contingency genes are localized to clusters of loci in facultative heterochromatin at subtelomeric and a few chromosome-internal sites. A limited number of these genes escape gene silencing and are expressed within the discontinuous stretches of facultative heterochromatin, with random switching between expressed members of multi-gene families conferring variant phenotypes on clonal progeny (62). Maintenance of a dynamic heterochromatin boundary is clearly critical to this process. In *S. cerevisiae,* H2A.Z antagonizes both the spread of Sir2-mediated silencing (42) and silencing of genes by the global sirtuin deacetylase Hst3, possibly through forming a localized barrier at promoters to Hst3 silencing activity (44). We have now shown that PfSir2A antagonizes the spread of total and acetylated Pf H2A.Z and Pf H2B.Z in an analogous process suggesting a role for H2A.Z as a heterochromatin barrier that is conserved across diverse taxa.

The best studied of the *P. falciparum* heterochromatic contingency genes is the *var* multigene family, a single member of which escapes silencing and is expressed in ring-stage parasites. We had previously shown that unlike other genes, silent *var* gene promoters were depleted of total Pf H2A.Z and Pf H2B.Z in wild-type *P. falciparum*. However, in *∆PfSir2A* parasites, multiple *var* genes are de-repressed, and Pf H2A.Z and Pf H2B.Z are enriched at their promoters (8, 36). Here, we show that Pf H2A.Z and Pf H2B.Z at these *var* promoters are acetylated. PfSir2A could directly deacetylate the small fraction of total Pf H2A.Z and Pf H2B.Z that are present at heterochromatin barriers and *var* promoters. Alternatively, PfSir2A could indirectly antagonize Pf H2A.Z/Pf H2B.Z and their acetylation through deacetylating other histones. In *S. cerevisiae,* the Swr1 complex is stably bound to heterochromatin boundaries and is induced to deposit H2A.Z/H2B dimers in promoter nucleosomes by the NuA4 histone acetyltransferase complex acetylating H4. Thus, acetylated H4 and H2A.Z coordinate to activate genes in these regions (43). *P. falciparum* has a *Swr1* ortholog, so PfSir2A could indirectly antagonize acetylated Pf H2A.Z and Pf H2B.Z by deacetylating H4 (63) and blocking Pf H2A.Z and Pf H2B.Z deposition by Swr1. In *S. cerevisiae,* the histone acetyltransferase GCN5 can acetylate H2A.Z (64), and the *P. falciparum* GCN5 bromodomain binds acetylated H4 (32). Consequently, by blocking PfGCN5 binding to H4ac sirtuins could also indirectly antagonize Pf H2A.Z and Pf H2B.Z acetylation.

We observed strong enrichment of acetylated Pf H2A.Z and Pf H2B.Z in *var* gene introns, which was associated with the introns’ promoter activity and exon 2 expression levels. Similarly, enrichment of homotypic H2A.Z nucleosomes at intron-exon boundaries in *Drosophila* was associated with gene expression and was proposed to be a consequence of the transcriptional machinery encountering regions of differing nucleosomal density (58). The enrichment at *var* intron boundaries could also possibly reflect a sequence preference for Pf H2A.Z- and Pf H2B.Z-containing nucleosomes as the *var* intron boundaries have a unique sequence composition and are enriched in complementary repeat sequences (65).

The differential enrichment of acetylated Pf H2A.Z and Pf H2B.Z in heterochromatin in *∆PfSir2A* parasites was often in the downstream sequences between convergent upsA and upsB *var* genes (Fig. 4 and 6; Fig. S3), suggesting the enrichment in this region was antagonized by PfSir2A. UpsB *var* genes are frequently the first coding sequence after non-coding subtelomeric repeats, so the enrichment of acetylated Pf H2A.Z and Pf H2B.Z in the *var* introns and downstream of the upsB *var* genes could play a fundamental role in containing the spread of silent, telomeric heterochromatin, rather than in *var* gene regulation *per se*. Alternatively, as the regions between upsA and upsB *var* genes are also enriched in other small contingency gene family members, e.g., *rif*, the enrichment of acetylated alternative histones here may relate to the variegated expression of members of these gene families.

In conclusion, the acetylated and total *P. falciparum* alternative histones Pf H2A.Z and Pf H2B.Z co-localize across the genome, suggesting that the majority of Pf H2A.Z and Pf H2B.Z are acetylated. However, their acetylation at gene promoters is dynamically associated with expression of stage-specific genes and *var* genes. These observations suggest they play an important role in marking promoters and in promoter activation. Acetylated Pf H2A.Z and Pf H2B.Z are also antagonized by PfSir2A and PfSir2B at heterochromatin boundaries, indicating they additionally are involved in maintaining proper chromatin structure in *P. falciparum*. Finally, they are enriched in *var* gene introns and downstream of mainly upsB *var* genes independent of *var* gene expression, suggesting they play a structural role in maintaining the chromatin compartments of *P. falciparum*.

## MATERIALS AND METHODS

### Parasites

The cloned PfSir2A and PfSir2B knockout made in a *P. falciparum* 3D7 clone background were previously described (36, 38) as was the wild-type 3C subpopulation of the *P. falciparum* 3D7 clone that was selected for predominantly *var2csa* expression by panning on purified chondroitin sulfate A (66). Parasite cultures were maintained as previously described (11) and regularly synchronized using 5% sorbitol.

### Histone Western blot

Pellets from 3D7 parasite cultures were lysed in 0.15% saponin in PBS, and the parasite pellets were extracted in 2× Laemmli buffer. Parasite extracts were loaded onto 12% BisTris gels (Invitrogen) and separated in 1× MES running buffer (Invitrogen). After transferring the proteins onto nitrocellulose membranes and blocking with 5% milk in Tris-buffered saline-Tween (TBS-T), the blot was cut into strips, and each strip was incubated with different antibodies overnight at a dilution of 1:2,000. After washing and incubation with horse radish peroxidase (HRP)-coupled goat anti-rabbit antibodies (Invitrogen), the strips were developed with Immobilon ECL reagent (Millipore). As all strips were derived from the same membrane, alignment of the strips facilitated accurate size determination of the detected histones and histone variants. Rabbit antibodies were anti-H3 (Abcam, Ab1790), anti-H4K12ac (Millipore 07-595), anti-H2B (Abcam, Ab1790). Images were acquired in the optimal dynamic range using the Vilber Fusion FX system.

### Dot blot

The peptides analyzed were the acetylated and non-acetylated version of the Pf H2A.Z and Pf H2B.Z peptides used for immunization (Fig. 1A) and H3K9ac ARTKQTARK(Ac)STAGKAPRKQLAGGK(Biot), H3K18ac ARTKQTARKSTAGKAPRK(Ac)QLAGGK(Biotin), H3K9K14K18ac ARTKQTARK(Ac)STAGK(Ac)APRK(Ac)QLAGGK(Biotin), H3K27ac ASKAARK(Ac)SAPISAGIKKPHRYRPGGK(Biot), H3K23K27ac ASK(Ac)AARK(Ac)SAPISAGIKKPHRYRPGGK(Biot), H3K56ac ALREIRRYQK(Ac)STDLLIRKLGGK(Biot), H4K5K8K12K16ac SGRG-K(Ac)-GG-K(Ac)-GLG-K(Ac)-GGA-K(Ac)-RHRKVLRDNGSGS-K(Biotin), H4K8ac SGRGKGG-K(Ac)-GLGKGGAKRHRKV-GGK(Biotin), H4K16ac SGRGKGGKGLGKGGA-K(Ac)-RHRKVGG-K(Biotin), H4K12ac SGRGKGGKGLG-K(Ac)-GGAKRHRKVGG-K(Biotin), and H4 SGRGAGGAGLGAGGAARHRKVLRDNGSGS-K(biotin). One microliter of peptides (200, 40, 8, and 1.6 ng) was spotted onto nitrocellulose membrane and air dried prior to blocking in Tris-buffered saline pH 7.5 0.05% Tween 20 and 5% (wt/vol) skim milk powder for at least 1 hour. For detection with antibodies, the membranes were then incubated overnight at 4°C with primary antibody in 5% skim milk in TBS-T α-Pf H2A.Zac 2 µg, α-Pf H2B.Zac 4 µg with rocking overnight. The next day, the membranes were washed three times in TBS-T for 15 minutes each at room temperature, incubated again with 5% skim milk in TBS-T for 30 minutes and then with 1/20,000 diluted goat α-rabbit-HRP (Invitrogen) in 5% skim milk in TBS-T, then washed three times for 20 minutes each in TBS-T, and then incubated for several minutes with Clarity enhanced chemiluminescence (ECL) reagents (Biorad) prior to imaging. For detection of biotinylated peptides, the blocked membrane was washed eight times for 10 minutes each in TBS-T, then incubated at room temperature for 30 minutes with 1/50,000 diluted streptavidin-HRP (Abcam) in TBS-T with rocking. The membrane was washed three times for 20 minutes each with TBS-T at 4°C with rocking prior to development with ECL reagent. For total peptide detection, an unblocked dotblot was incubated with 0.1% (wt/vol) Ponceau S in 5% acetic acid for 5 minutes and destained in water.

### HDAC inhibitor assay

Wild-type parasite cultures were tightly synchronized and seeded in triplicates into six-well plates at 7% ring-stage parasitemia. Cultures were treated with 200 nM Trichostatin A (TSA) or 70 nM apicidine and equivalent volumes of dimethyl sulfoxide (DMSO) as a control. The cultures were incubated for 4 hours with the inhibitors before red blood cells were lysed with 0.15% saponin in PBS, and parasite pellets were extracted for Western blot (67). Parasite proteins were separated by SDS-PAGE on 4%–12% Bis-Tris gels (Invitrogen) and transferred to nitrocellulose membranes. The membranes were blocked with 5% non-fat milk in TBS-T buffer and incubated overnight with primary antibodies at a dilution of 1:2,000. Secondary goat anti-rabbit HRP antibodies were used at a dilution of 1:20,000.

### Cross-linked chromatin immunoprecipitation

Synchronized PfSir2A-KO and wild-type parasites were harvested at ring stage 16 hours post invasion and at schizont stage 42 hours post invasion and cross-linked chromatin prepared as described previously (8). Sonicated chromatin was diluted 1:10 in ChIP dilution buffer (0.01% SDS, 1.1% Triton X-100, 1.2 mM EDTA, 16.7 mM Tris-HCl pH 8.1, 150 mM NaCl) and mixed with an equal volume of Protein A/Protein G Sepharose beads (GE Healthcare). Antibodies used were rabbit anti-Pf H2A.Z and its preimmune serum (8), and the anti-Pf H2A.Zac, anti-Pf H2B.Zac, and pan-reactive anti-Pf H2B.Z generated by Anaspec in rabbits as described in the Results, rabbit anti-H3K9me3 (Active Motif catalog number 39161), a rabbit IgG polyclonal isotype control (Abcam ab37415) non-specific antibody was used as a negative control for the ChIPs with Anaspec antibodies. Chromatin was immunoprecipitated overnight at 4°C, and then the beads were serially washed for 5 minutes with agitation at 4°C first with 1 mL of low salt immune complex wash buffer (0.1% SDS, 1% Triton X-100, 2 mM EDTA, 20 mM Tris-HCl pH 8.1, 150 mM NaCl), then with high salt immune complex wash buffer (0.1% SDS, 1% Triton X-100, 2 mM EDTA, 20 mM Tris-HCl pH 8.1, 500 mM NaCl), then with LiCl immune complex wash buffer (0.25M LiCl, 1% IGEPAL-630, 1% deoxycholate, 1 mM EDTA, 10 mM Tris-HCl pH 8.1). Beads were then washed twice at room temperature for 5 minutes each with Tris-EDTA buffer (10 mM Tris-HCl pH 8.1, 1 mM EDTA pH 8.0) and then chromatin eluted by incubating twice with separate 100 µL aliquots of freshly made elution buffer (1% SDS, 0.1 M NaHCO_3_) for 15 minutes at room temperature.

### ChIP-seq and RNA-seq library preparation

ChIP DNA samples were quantified with the Qubit dsDNA HS Assay Kit (Q32851) (Invitrogen), and 4 ng of each ChIP DNA was used to make sequencing libraries with the NEBNext Ultra II DNA Library Prep Kit for Illumina (E7645) but using KAPA HiFi PCR kit containing high-fidelity KAPA HiFi DNA polymerase enzyme (Roche) for library amplification (12 cycles of 10 s at 98°C and 1 minute at 65°C).

RNA from ring-stage- and schizont-stage-infected erythrocytes was extracted and used to make Illumina RNAseq libraries as previously described (68) but with the omission of globin mRNA depletion.

ChIP DNA and cDNA libraries were quantitated and checked for adapter ligation by qPCR using P5/P7 primers, and library size, purity, and quality were confirmed with the Agilent High Sensitivity DNA kit and reagents (Agilent) on the automated electrophoresis Agilent 2100 Bioanalyzer system. The 150-bp paired-end libraries were sequenced on the Illumina Novaseq 6000 system at the Victorian Clinical Genetics Services, Murdoch’s Children Research Institute, Parkville, Victoria, Australia.

### Sequence pre-processing

Fastq files were checked for quality using FastQC tool (version 0.11.8, https://www.bioinformatics.babraham.ac.uk/projects/fastqc/), and adaptor sequences were trimmed with TrimGalore (version 0.4.4, www.bioinformatics.babraham.ac.uk/projects/trim_galore).

### RNA-seq analysis

Trimmed reads were aligned to the *P. falciparum* 3D7 reference genome (version 34) using STAR (version 2.6.0) (69) and converted to BAM format, sorted, and indexed using SAMtools version 1.9 (70). Gene read counts were determined from BAM files using featureCounts (71) and counts normalized by expressing as fragments per kilobase of exon per million reads mapped (FPKM) or reads per genomic content [reads normalized to 1× sequencing depth where sequencing depth is defined as: (mapped reads x fragment length)/23 Mb genome size]. FPKM values were used to rank genes by expression.

### ChIP-seq analysis

Trimmed reads were aligned to the *P. falciparum* 3D7 reference genome, version 34 (PlasmoDB) using bowtie2 version 2.3.4.3 (72). Alignments were converted to BAM format, sorted, and indexed using SAMtools version 1.9 (70), and the indexed and sorted bam files were converted into bigwig formatted files using the bamcoverage tool with a 50-bp window from the Deeptools suite (73). Peaks of ChIP library reads that were enriched relative to input library reads were called using MACS2 version 2.1.2.1 (49), with the *q*-value threshold parameter of 0.05. Called peaks were visualized with the Integrated Genome Viewer (74). ChIP peaks that were differentially enriched between different ChIP libraries were identified using CSAW (50) with the default false discovery rate of 0.05.

### Analysis of ChIP read counts within boundaries between euchromatin and H3K9me3-enriched heterochromatin

MACS2 (49)-called peaks of H3K9me3/input in 3D7 parasites were used to identify regions of heterochromatin in ring and schizont stages. Read counts were determined using featureCounts (71) for Pf H2A.Z, Pf H2A.Zac, Pf H2B.Z, and Pf H2B.Zac within heterochromatin and for 10 kb of flanking sequence on each side of the heterochromatic regions. In cases where two heterochromatic regions were separated by less than 10 kb, the entire interval between them was used. In cases where flanking sequences completely overlapped, one was randomly chosen for analysis. Of the 100–130 regions analyzed, eight flanking sequences partially overlapped and were retained. Bedtools shuffle (75) was used to randomly select size-matched control sequences from the same chromosome as the heterochromatic and flanking sequences with heterochromatic and flanking sequences excluded. Heterochromatic and flanking region read-depth was normalized as RPKM, which was converted to a ratio of log_2_(ChIP/input) and averaged for the two replicates. The read count ratios were compared between wild-type and *∆PfSir2A* samples using a Wilcoxon matched-pairs signed-rank test.

## Data Availability

All sequences are available as BioProject PRJNA1023675.

## SUPPLEMENTAL MATERIAL

Fig. S1 mbio.02014-23-s0001.pdf Anti-Pf H2A.Zac and anti-Pf H2B.Zac do not bind acetylated H3 and H4 peptides.

Fig. S2 mbio.02014-23-s0002.pdf Spearman correlation heatmaps.

Fig. S3 mbio.02014-23-s0003.pdf Total and acetylated Pf H2A.Z and Pf H2B.Z enrichment in heterochromatin.

Fig. S4 mbio.02014-23-s0004.pdf Total and acetylated Pf H2A.Z and Pf H2B.Z are antagonized by PfSir2B at heterochromatin boundaries.

Fig. S5 mbio.02014-23-s0005.pdf Pf H2A.Z and Pf H2B.Z are enriched at transcribed *var* gene promoters and *var* introns.

Fig. S6 mbio.02014-23-s0006.pdf Pf H2A.Z is deacetylated by class I and II HDACs.

ASM does not own the copyrights to Supplemental Material that may be linked to, or accessed through, an article. The authors have granted ASM a non-exclusive, world-wide license to publish the Supplemental Material files. Please contact the corresponding author directly for reuse.
