# Supplementary material for: The P. falciparum alternative histones Pf H2A.Z and Pf H2B.Z are dynamically acetylated and antagonized by PfSir2 histone deacetylases at heterochromatin boundaries
Source: mBio. 2023 Oct 26;14(6):e02014-23. doi: 10.1128/mbio.02014-23 (PMC10746207; doi:10.1128/mbio.02014-23)
Supplement: Fig. S1 — Anti-Pf H2A.Zac and anti-Pf H2B.Zac do not bind acetylated H3 and H4 peptides. [file mbio.02014-23-s0001.pdf]

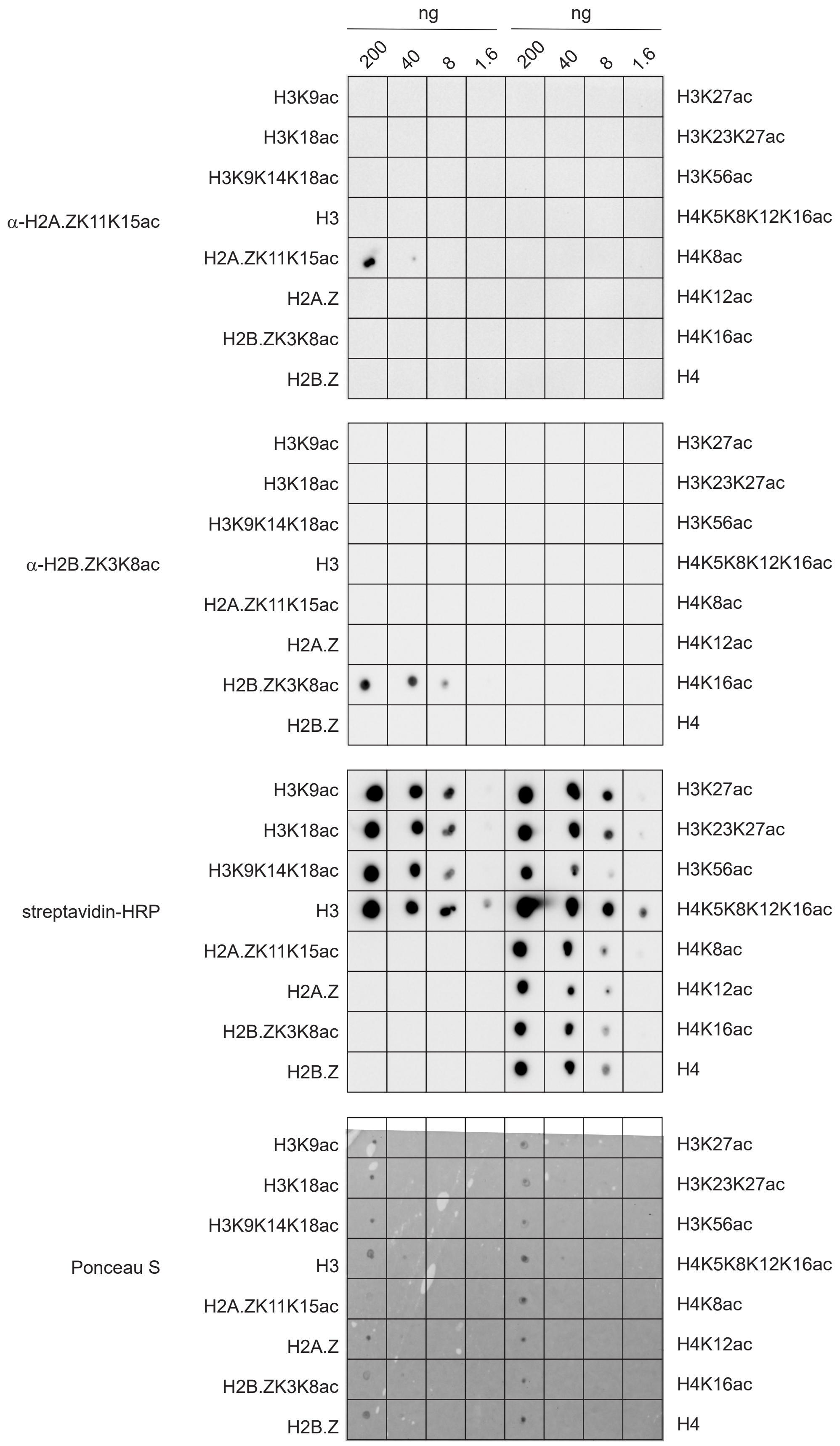


**Suppl Fig 1. Anti-Pf H2A.Zac and anti-Pf H2B.Zac do not bind acetylated H3 and H4 peptides**. Peptide quantities indicated at the top of the figure were dotted onto nitrocellulose and the dotblots were probed either with the antibodies indicated to the left or with streptavidin-HRP. The Pf H2A.Z and Pf H2B.Z unmodified and acetylated peptides were not biotinylated and so did not bind the streptavidin-HRP. Equivalent loading of the different peptides was confirmed by staining a dotblot with Ponceau S (bottom).
