# Supplementary figures and images for: The P. falciparum alternative histones Pf H2A.Z and Pf H2B.Z are dynamically acetylated and antagonized by PfSir2 histone deacetylases at heterochromatin boundaries
Source: mBio. 2023 Oct 26;14(6):e02014-23. doi: 10.1128/mbio.02014-23 (PMC10746207; doi:10.1128/mbio.02014-23)

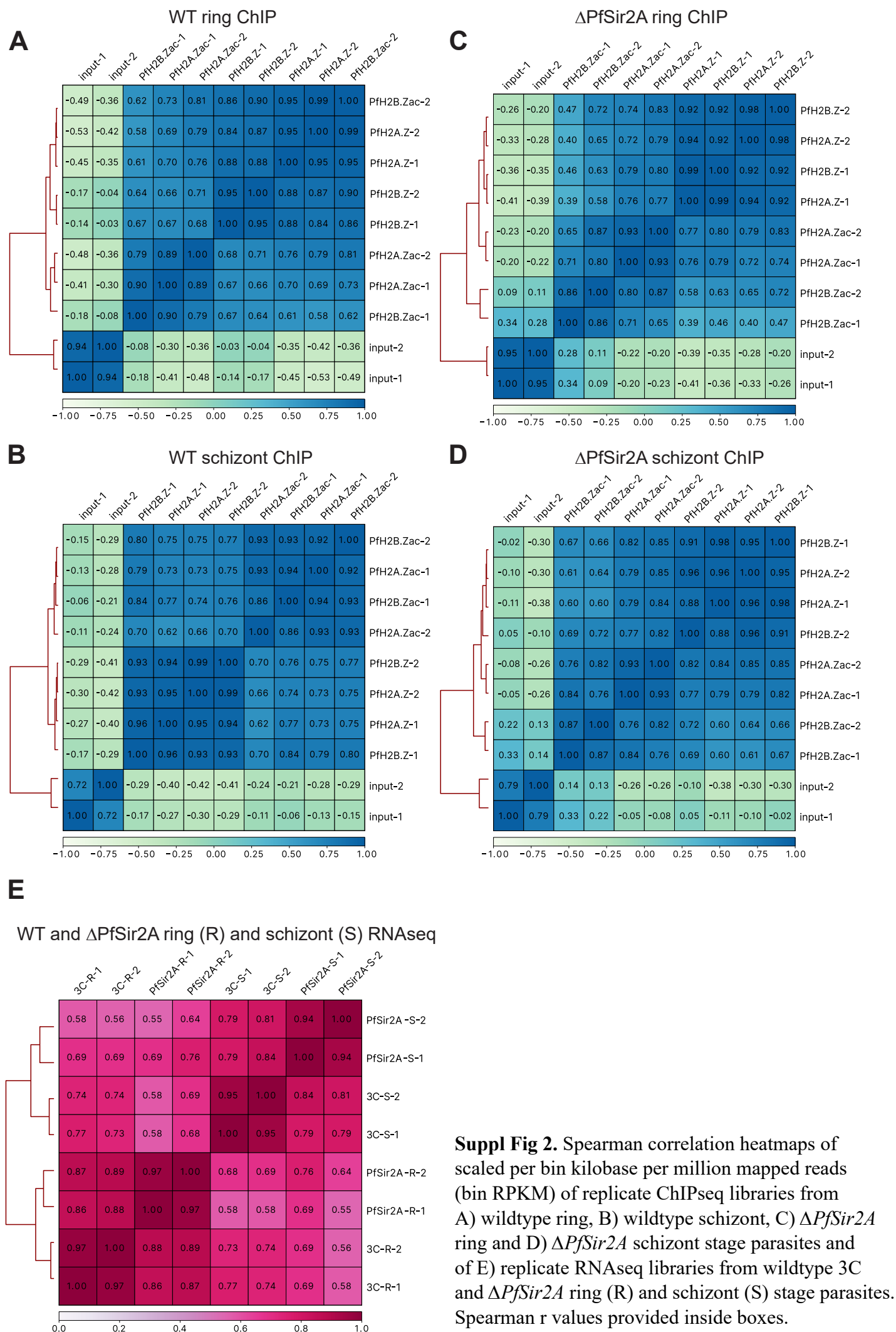

Supplement: Fig. S2 — Spearman correlation heatmaps. [file mbio.02014-23-s0002.pdf]
