# Supplementary material for: The P. falciparum alternative histones Pf H2A.Z and Pf H2B.Z are dynamically acetylated and antagonized by PfSir2 histone deacetylases at heterochromatin boundaries
Source: mBio. 2023 Oct 26;14(6):e02014-23. doi: 10.1128/mbio.02014-23 (PMC10746207; doi:10.1128/mbio.02014-23)
Supplement: Fig. S3 — Total and acetylated Pf H2A.Z and Pf H2B.Z enrichment in heterochromatin. [file mbio.02014-23-s0003.pdf]

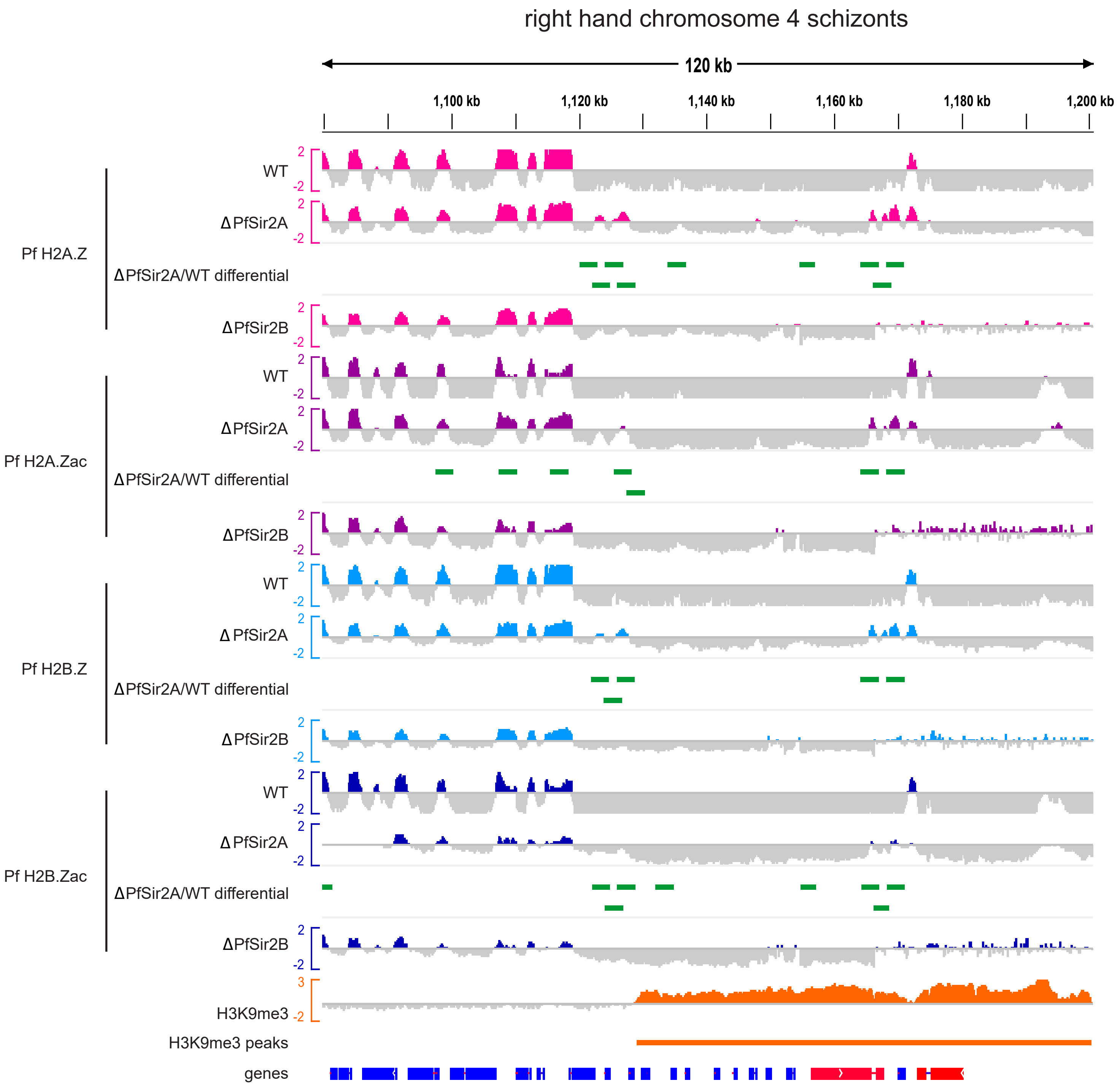

Suppl Fig 3. Total and acetylated Pf H2A.Z and Pf H2B.Z are differentially enriched in heterochromatin in Δ*PfSir2A and* Δ*PfSir2B* compared to wildtype schizonts. A projection of 111 kb from the right-hand end of chromosome 4 in schizont stage wildtype, Δ*PfSir2A,* and Δ*PfSir2B* parasites showing log2(ChIP/input) of Pf H2A.Z, Pf H2B.Z, Pf H2A.Zac and Pf H2B.Zac and peaks of each total and acetylated histone that were identified as differentially enriched in Δ*PfSir2A* compared to wildtype schizonts (green); log2(ChIP/input) of H3K9me3; and peaks of H3K9me3 in wildtype 3D7 schizonts (orange); all genes in blue with *var* genes superimposed in red.
