# Supplementary material for: The P. falciparum alternative histones Pf H2A.Z and Pf H2B.Z are dynamically acetylated and antagonized by PfSir2 histone deacetylases at heterochromatin boundaries
Source: mBio. 2023 Oct 26;14(6):e02014-23. doi: 10.1128/mbio.02014-23 (PMC10746207; doi:10.1128/mbio.02014-23)
Supplement: Fig. S4 — Total and acetylated Pf H2A.Z and Pf H2B.Z are antagonized by PfSir2B at heterochromatin boundaries. [file mbio.02014-23-s0004.pdf]

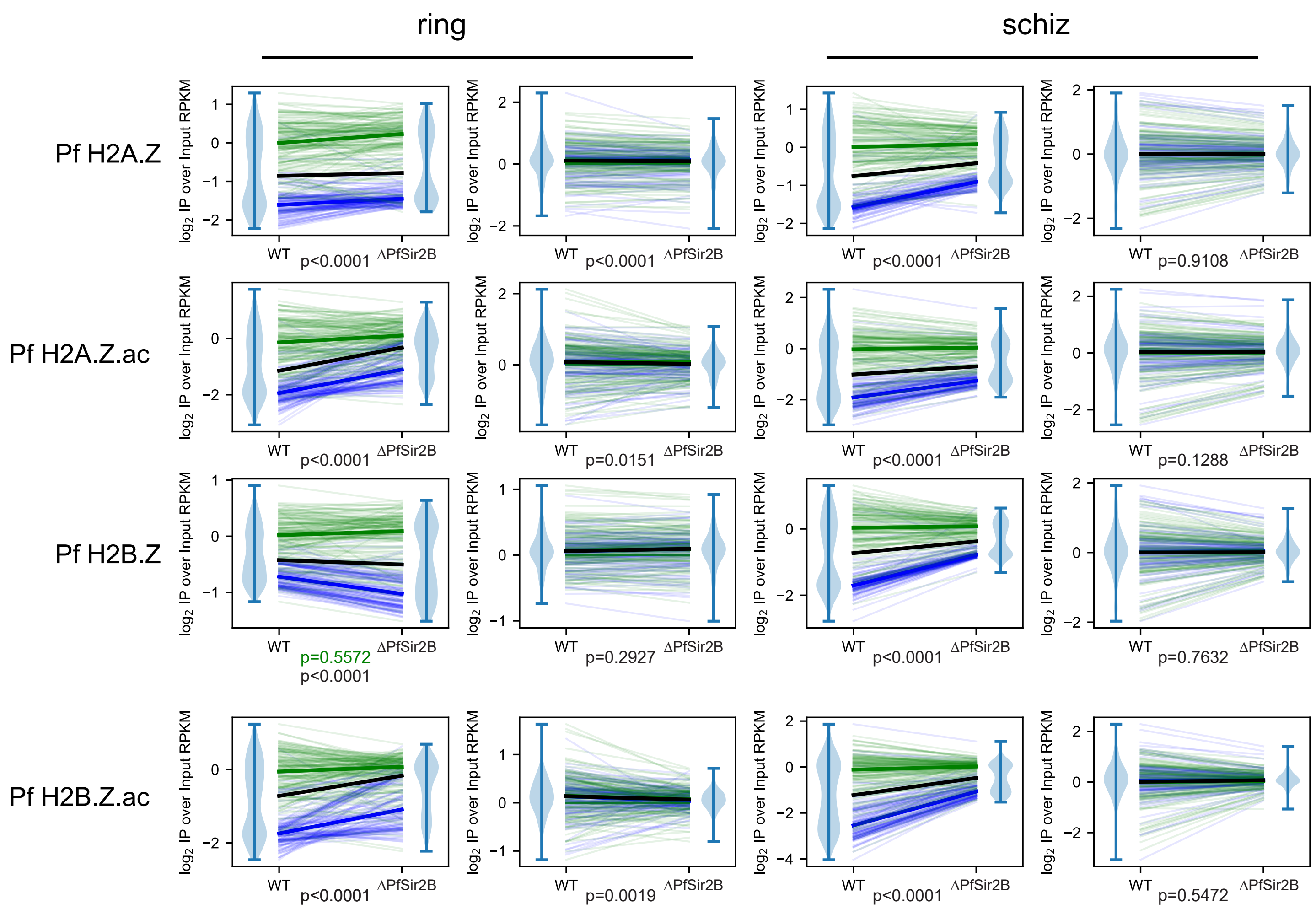

Suppl Fig 4. Total and acetylated Pf H2A.Z and Pf H2B.Z are antagonised by PfSir2B at heterochromatin boundaries. Average ratios of ChIP/input for two replicates of total and acetylated Pf H2A.Z and Pf H2B.Z ChIP normalised as Reads per kilobase per million reads (RPKM) from wildtype (WT) compared to a single replicate of Δ*PfSir2B* parasites. The test and control regions were the same as used in Fig 5. Medians for heterochromatin and matched controls (blue) or flanks and matched controls (green) are shown in thick, bold lines and medians for the combined heterochromatin and flank test regions or their matching controls are shown in thick black bold. P values are for Wilcoxon matched-pairs signed rank test comparisons between wildtype and Δ*PfSir2B* parasites of combined heterochromatin and flank test regions or their matching controls (in black) or flanking regions only (in green for Pf H2B.Z). Violin plots at the edges of line plots indicate the frequency distribution of the combined heterochromatin and flank regions or matching control regions and bars indicate the range.
