# Supplementary material for: The P. falciparum alternative histones Pf H2A.Z and Pf H2B.Z are dynamically acetylated and antagonized by PfSir2 histone deacetylases at heterochromatin boundaries
Source: mBio. 2023 Oct 26;14(6):e02014-23. doi: 10.1128/mbio.02014-23 (PMC10746207; doi:10.1128/mbio.02014-23)
Supplement: Fig. S5 — Pf H2A.Z and Pf H2B.Z are enriched at transcribed var gene promoters and var introns. [file mbio.02014-23-s0005.pdf]

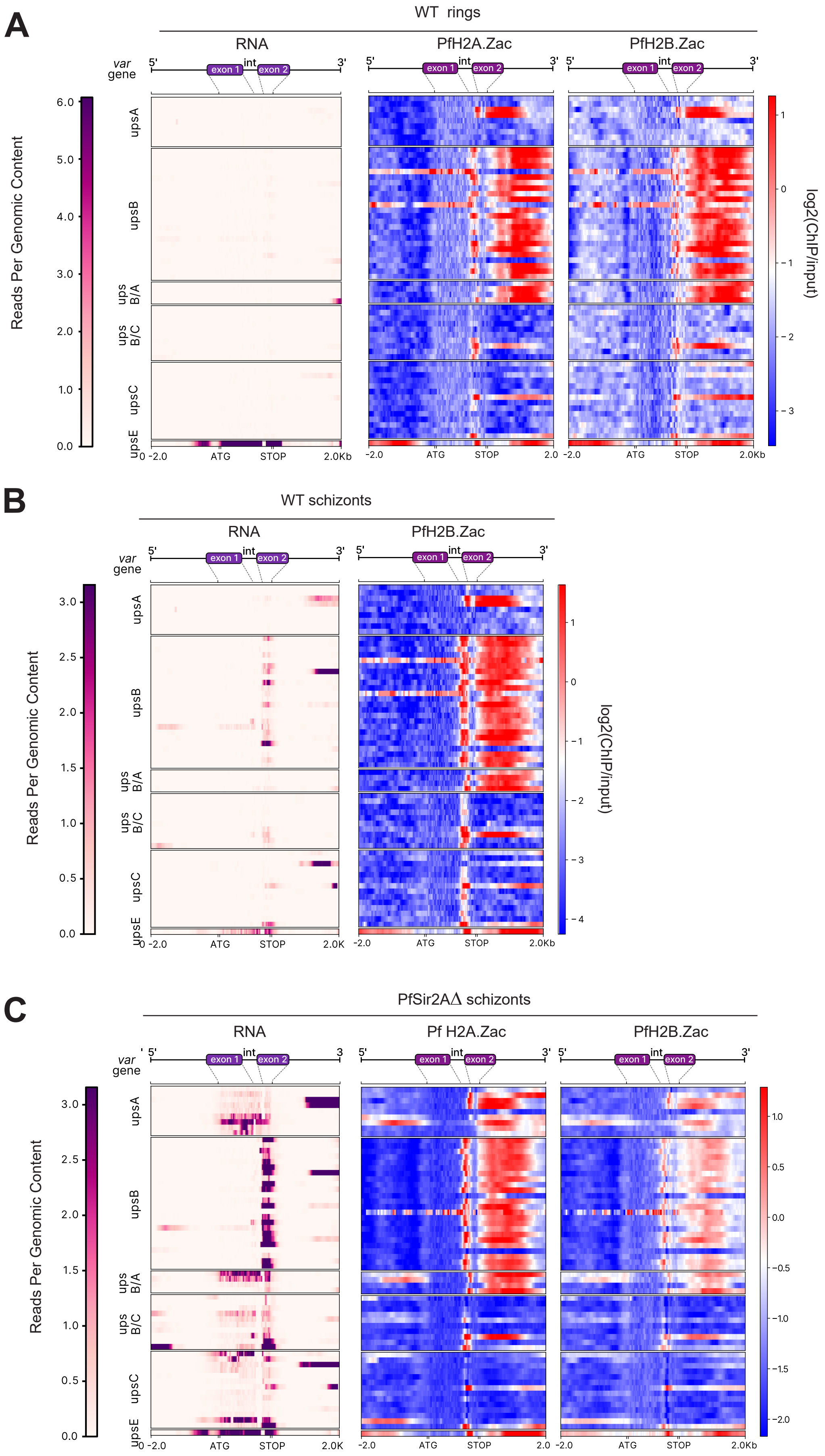

Suppl Fig 5. Pf H2A.Z and Pf H2B.Z are enriched at transcribed *var* gene promoters and *var* introns. Complement of data in Fig 6 showing RNAseq RPGC) and log2(ChIP/input) for total and acetylated Pf H2A.Z and Pf H2B.Z plotted across the genomic repertoire of 65 *var* genes in A) ring stage and B) schizont stage wildtype and C) Δ*PfSir2A* parasites. The heatmaps are scaled to render all *var* genes the same length, therefore the gene diagrams at the top of the heatmaps only approximate the intron-exon boundaries (intron; int).
