# Supplementary material for: The P. falciparum alternative histones Pf H2A.Z and Pf H2B.Z are dynamically acetylated and antagonized by PfSir2 histone deacetylases at heterochromatin boundaries
Source: mBio. 2023 Oct 26;14(6):e02014-23. doi: 10.1128/mbio.02014-23 (PMC10746207; doi:10.1128/mbio.02014-23)
Supplement: Fig. S6 — Pf H2A.Z is deacetylated by class I and II HDACs. [file mbio.02014-23-s0006.pdf]

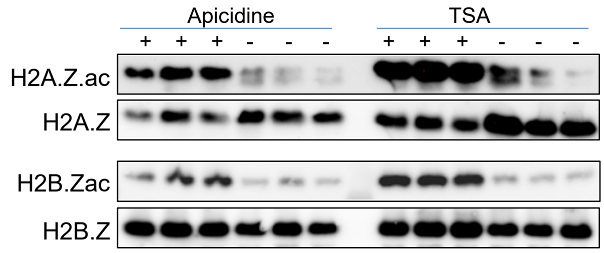

Suppl Fig 6. Pf H2A.Z is deacetylated by class I and II histone deacetylases (HDACs). 3D7 parasites were grown in the presence (+) or absence (-) of the class I and II HDAC inhibitors apicidin (Api) and trichostatin A (TSA) for <4 hours (h) and then schizonts released by saponin lysis of infected erythrocytes were probed on western blot with antibodies to total Pf H2A.Z total Pf H2B.Z, Pf H2A.Zac or Pf H2B.Zac.
